# (4*Z*,6*Z*)-4,6-Bis(4-meth­oxy­benzyl­idene)-2,2-dimethyl-1,3-dioxan-5-one

**DOI:** 10.1107/S1600536812000372

**Published:** 2012-01-11

**Authors:** Mohammad M. Mojtahedi, Werner Massa, M. Saeed Abaee, A. Wahid Mesbah

**Affiliations:** a Organic Chemistry Laboratory, Chemistry and Chemical Engineering, Research Center of Iran, PO Box 14335-186, Tehran, Iran; b Fachbereich Chemie der Philipps-Universität, Hans-Meerwein-Strasse, D-35043 Marburg, Germany

## Abstract

The title compound, C_22_H_22_O_5_, crystallizes with two independent mol­ecules in the asymmetric unit, both of which possess pseudo-*C*_s_ symmetry. The central 1,3-dioxanone rings have envelope conformations, with the C atom bearing the two methyl groups at the flap. The benzene rings of the meth­oxy­benzyl­idene units, attached in the 4- and 6-positions on the central 1,3-dioxanone rings, are tilted in the same direction with dihedral angles varying between 8.2 (1) and 18.1 (1)°. The crystal packing is influenced by π-stacking inter­actions of the parallel displaced type [centroid–centroid distance of 3.723 (1) Å for mol­ecule 1 and 3.884 (1) Å for mol­ecule 2, with ring slippages of 1.432 and 1.613 Å, respectively] and the T-shaped type, with the long mol­ecular axes all aligned along [010].

## Related literature

For the synthesis of bis­aryl­idenes of hetero- and homocyclic ketones, see: Abaee *et al.* (2008*a* ▶,*b* ▶). For the crystal structures of similar compounds, see: Abaee *et al.* (2012 ▶); Nesterov *et al.* (2011 ▶); Shahani *et al.* (2010 ▶). For details concerning π-stacking inter­actions, see: Hunter & Sanders (1990 ▶).
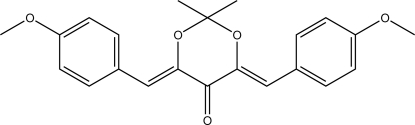


## Experimental

### 

#### Crystal data

C_22_H_22_O_5_
*M* *_r_* = 366.39
Monoclinic, 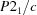

*a* = 9.2400 (7) Å
*b* = 40.384 (4) Å
*c* = 10.1643 (8) Å
β = 91.988 (9)°
*V* = 3790.5 (6) Å^3^
*Z* = 8
Mo *K*α radiation
μ = 0.09 mm^−1^
*T* = 193 K
0.45 × 0.24 × 0.03 mm

#### Data collection

Stoe IPDS diffractometer
Absorption correction: multi-scan (Blessing, 1995 ▶) *T*_min_ = 0.972, *T*_max_ = 1.000
28928 measured reflections
6486 independent reflections
3306 reflections with *I* > 2σ(*I*)
*R*_int_ = 0.068

#### Refinement

*R*[*F* ^2^ > 2σ(*F* ^2^)] = 0.034
*wR*(*F* ^2^) = 0.058
*S* = 0.87
6486 reflections
495 parameters
H-atom parameters constrained
Δρ_max_ = 0.13 e Å^−3^
Δρ_min_ = −0.15 e Å^−3^

### 

Data collection: *EXPOSE* (Stoe & Cie, 1999 ▶); cell refinement: *CELL* (Stoe & Cie, 1999 ▶); data reduction: *INTEGRATE* (Stoe & Cie, 1999 ▶); program(s) used to solve structure: *SHELXS97* (Sheldrick, 2008 ▶); program(s) used to refine structure: *SHELXL97* (Sheldrick, 2008 ▶); molecular graphics: *DIAMOND* (Brandenburg, 2011 ▶); software used to prepare material for publication: *publCIF* (Westrip, 2010 ▶).

## Supplementary Material

Crystal structure: contains datablock(s) I, global. DOI: 10.1107/S1600536812000372/su2363sup1.cif

Structure factors: contains datablock(s) I. DOI: 10.1107/S1600536812000372/su2363Isup2.hkl

Supplementary material file. DOI: 10.1107/S1600536812000372/su2363Isup3.cml

Additional supplementary materials:  crystallographic information; 3D view; checkCIF report

## Figures and Tables

**Table 1:** Hydrogen-bond geometry (Å, °) *Cg*(II_2) is the centroid of ring II (C8–C13) of mol­ecule 2.

*D*—H⋯*A*	*D*—H	H⋯*A*	*D*⋯*A*	*D*—H⋯*A*
C13—H13⋯*Cg*(II_2)^i^	0.95	2.68	3.604 (2)	164

Symmetry code: (i) 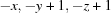.

**Fig. 1.**
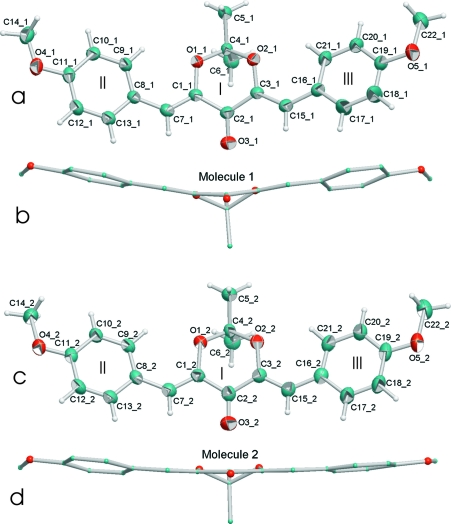
A view of the two independent molecules of the title compound, with atom numbering and 50% probability displacement ellipsoids (a,c); schematic views of the two independent molecules (omitting H atoms) approximately along the central ring mean plane [O1,C1-C3,O2] (b,d).

**Fig. 2.**
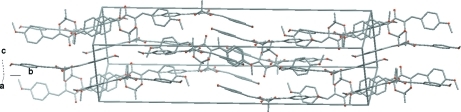
Crystal packing of the title compound [O atoms red; H atoms omitted for clarity].

## supplementary crystallographic information

### Comment

In the course of our investigations on the synthesis of bisarylidenes of hetero- and homo-cyclic ketones (Abaee *et al.*, 2008*a*,*b*), we herein report on the synthesis and crystal structure of the title compound.

The asymmetric unit of the title compound contains two crystallographically independent molecules (1 and 2, Fig. 1), both having pseudo-C_s_ symmetry. The bond distances and angles are close to those observed in similar compounds (Abaee *et al.*, 2012; Nesterov *et al.*, 2011; Shahani *et al.*, 2010).

The two independent molecules differ mainly in the degree of bending of the benzene substituents with respect to the almost planar part of the central 1,3-dioxanone ring [plane I: (O1,C1-C3,O2); max. deviation 0.0584 (18) Å in molecule 1, and 0.0166 (18) Å in molecule 2], as shown in Fig. 1. The dihedral angles between this mean plane and the benzene rings, II [C8–C13] and III [C16–C21], are respectively, 17.4 (1) and 18.1 (1)° for molecule 1, and 15.7 (1) and 8.2 (1)° for molecule 2. The benzene rings are inclined to one another by 24.8 (1) in molecule 1, and 13.7 (1)° in molecule 2. Thus, molecule 2 is closer to planarity than molecule 1.

The crystal packing is influenced by π-stacking interactions (Hunter & Sanders, 1990) in a parallel displaced way concerning benzene ring II (C8–C13) and its symmetry equivalent in both independent molecules [symmetry center: -*x*, -*y*, -*z+1* for molecule 1, and -*x+1*, -*y+2*, -*z+2* for molecule 2]. The centroid-centroid distances are 3.723 (1) Å for molecule 1, and 3.884 (1) Å for molecule 2, with ring slippages of 1.432 and 1.613 Å, respectively. In addition, a T-shaped π-stacking contact is observed, involving the same benzene ring, II of molecule 1, *via* a C–H···π interaction with an equivalent benzene ring II of molecule 2 (Table 1). These interactions result in a mutually perpendicular orientation of molecules 1 and 2, and a parallel orientation of the benzene rings II of all molecules of type 1 to each other and all molecules of type 2 to each other. All the long axes of both molecules are oriented parallel to the [010] direction (Fig. 2).

### Experimental

A mixture of 2,2-dimethyl-1,3-dioxan-5-one (2 mmol), 4-methoxybenzaldehyde (4 mmol), diethylamine (8 mmol), and MgBr_2_.OEt_2_ (0.2 mmol, 10 mol%) was stirred at room temperature under an atmosphere of argon for 2 h. The progress of the reaction was checked by TLC using a 1:4 mixture of EtOAc/hexane. At the end of the reaction, the mixture was diluted by CH_2_Cl_2_ and washed with brine. The organic layer was dried using Na_2_SO_4_ and concentrated under reduced pressure. The product was isolated (83%) by column chromatography over silicagel using a 1:4 mixture of EtOAc/hexane. The solid product was recrystallized from EtOAc, giving yellow plate-like crystals of the title compound.

### Refinement

All the H atoms could be located in a difference Fourier map. In the final cycles of refinement they were included in calculated positions and treated as riding atoms: C–H = 0.95 and 0.98 Å for CH and CH_3_ H-atoms, respectively, with *U*_iso_(H) = k *x U*_eq_(parent C-atom), where k = 1.5 for CH_3_ H-atoms and k = 1.2 for all other H-atoms.

### Crystal data

**Table:**

C_22_H_22_O_5_	*F*(000) = 1552
*M**_r_* = 366.39	*D*_x_ = 1.284 Mg m^−^^3^
Monoclinic, *P*2_1_/*c*	Mo *K*α radiation, λ = 0.71073 Å
Hall symbol: -P 2ybc	Cell parameters from 8001 reflections
*a* = 9.2400 (7) Å	θ = 2.0–25.9°
*b* = 40.384 (4) Å	µ = 0.09 mm^−^^1^
*c* = 10.1643 (8) Å	*T* = 193 K
β = 91.988 (9)°	Platelet, yellow
*V* = 3790.5 (6) Å^3^	0.45 × 0.24 × 0.03 mm
*Z* = 8	

### Data collection

**Table:**

Stoe IPDS diffractometer	6486 independent reflections
Radiation source: fine-focus sealed tube	3306 reflections with *I* > 2σ(*I*)
graphite	*R*_int_ = 0.068
Detector resolution: 6.7 pixels mm^-1^	θ_max_ = 25.0°, θ_min_ = 2.1°
φ–scans	*h* = −10→10
Absorption correction: multi-scan (Blessing, 1995)	*k* = −48→48
*T*_min_ = 0.972, *T*_max_ = 1.000	*l* = −12→12
28928 measured reflections	

### Refinement

**Table:**

Refinement on *F*^2^	Primary atom site location: structure-invariant direct methods
Least-squares matrix: full	Secondary atom site location: difference Fourier map
*R*[*F*^2^ > 2σ(*F*^2^)] = 0.034	Hydrogen site location: inferred from neighbouring sites
*wR*(*F*^2^) = 0.058	H-atom parameters constrained
*S* = 0.87	*w* = 1/[σ^2^(*F*_o_^2^) + (0.010*P*)^2^] where *P* = (*F*_o_^2^ + 2*F*_c_^2^)/3
6486 reflections	(Δ/σ)_max_ = 0.001
495 parameters	Δρ_max_ = 0.13 e Å^−^^3^
0 restraints	Δρ_min_ = −0.15 e Å^−^^3^

### Special details

**Table:**

Geometry. All e.s.d.'s (except the e.s.d. in the dihedral angle between two l.s. planes) are estimated using the full covariance matrix. The cell e.s.d.'s are taken into account individually in the estimation of e.s.d.'s in distances, angles and torsion angles; correlations between e.s.d.'s in cell parameters are only used when they are defined by crystal symmetry. An approximate (isotropic) treatment of cell e.s.d.'s is used for estimating e.s.d.'s involving l.s. planes.
Refinement. Refinement of *F*^2^ against ALL reflections. The weighted *R*-factor *wR* and goodness of fit *S* are based on *F*^2^, conventional *R*-factors *R* are based on *F*, with *F* set to zero for negative *F*^2^. The threshold expression of *F*^2^ > σ(*F*^2^) is used only for calculating *R*-factors(gt) *etc*. and is not relevant to the choice of reflections for refinement. *R*-factors based on *F*^2^ are statistically about twice as large as those based on *F*, and *R*- factors based on ALL data will be even larger.

### Fractional atomic coordinates and isotropic or equivalent isotropic displacement parameters (Å^2^)

**Table:**

	*x*	*y*	*z*	*U*_iso_*/*U*_eq_	
O1_1	0.13652 (14)	0.09396 (3)	0.47980 (12)	0.0350 (3)	
O2_1	0.11486 (14)	0.15136 (3)	0.46692 (12)	0.0373 (3)	
O3_1	−0.20552 (17)	0.11456 (3)	0.32822 (14)	0.0513 (4)	
O4_1	0.27071 (16)	−0.06434 (3)	0.36675 (13)	0.0474 (4)	
O5_1	0.06933 (19)	0.31337 (3)	0.40567 (16)	0.0695 (5)	
C1_1	0.0103 (2)	0.08847 (4)	0.40709 (17)	0.0312 (5)	
C2_1	−0.0809 (2)	0.11758 (4)	0.37529 (18)	0.0345 (5)	
C3_1	−0.0170 (2)	0.15054 (4)	0.39966 (18)	0.0327 (5)	
C4_1	0.1442 (2)	0.12406 (4)	0.55320 (17)	0.0318 (5)	
C5_1	0.2989 (2)	0.12787 (5)	0.5977 (2)	0.0465 (5)	
H5A_1	0.3605	0.1273	0.5212	0.070*	
H5B_1	0.3261	0.1097	0.6578	0.070*	
H5C_1	0.3118	0.1491	0.6435	0.070*	
C6_1	0.0396 (2)	0.12348 (5)	0.66347 (18)	0.0452 (6)	
H6A_1	−0.0584	0.1193	0.6274	0.068*	
H6B_1	0.0420	0.1449	0.7090	0.068*	
H6C_1	0.0672	0.1059	0.7258	0.068*	
C7_1	−0.0214 (2)	0.05840 (4)	0.35759 (17)	0.0338 (5)	
H7_1	−0.1112	0.0570	0.3095	0.041*	
C8_1	0.0604 (2)	0.02747 (4)	0.36605 (17)	0.0313 (5)	
C9_1	0.1886 (2)	0.02302 (4)	0.43971 (19)	0.0377 (5)	
H9_1	0.2276	0.0411	0.4894	0.045*	
C10_1	0.2614 (2)	−0.00718 (4)	0.44252 (19)	0.0391 (5)	
H10_1	0.3486	−0.0096	0.4940	0.047*	
C11_1	0.2071 (2)	−0.03357 (4)	0.37075 (19)	0.0361 (5)	
C12_1	0.0799 (2)	−0.02982 (5)	0.29576 (19)	0.0410 (5)	
H12_1	0.0423	−0.0478	0.2451	0.049*	
C13_1	0.0080 (2)	0.00014 (4)	0.29488 (18)	0.0374 (5)	
H13_1	−0.0800	0.0022	0.2443	0.045*	
C14_1	0.3863 (2)	−0.07054 (5)	0.4593 (2)	0.0530 (6)	
H14A_1	0.4149	−0.0939	0.4545	0.079*	
H14B_1	0.3547	−0.0656	0.5482	0.079*	
H14C_1	0.4691	−0.0565	0.4392	0.079*	
C15_1	−0.0773 (2)	0.17839 (4)	0.35254 (19)	0.0393 (5)	
H15_1	−0.1626	0.1754	0.2990	0.047*	
C16_1	−0.0316 (2)	0.21270 (5)	0.37103 (19)	0.0392 (5)	
C17_1	−0.0991 (3)	0.23727 (5)	0.2980 (3)	0.0814 (9)	
H17_1	−0.1731	0.2312	0.2354	0.098*	
C18_1	−0.0641 (3)	0.27025 (6)	0.3117 (3)	0.0900 (10)	
H18_1	−0.1133	0.2863	0.2586	0.108*	
C19_1	0.0407 (2)	0.27998 (5)	0.4012 (2)	0.0472 (6)	
C20_1	0.1084 (2)	0.25658 (5)	0.4774 (2)	0.0513 (6)	
H20_1	0.1803	0.2630	0.5416	0.062*	
C21_1	0.0729 (2)	0.22334 (5)	0.4616 (2)	0.0487 (6)	
H21_1	0.1223	0.2074	0.5150	0.058*	
C22_1	0.1718 (3)	0.32471 (5)	0.5016 (3)	0.0716 (8)	
H22A_1	0.1857	0.3486	0.4913	0.107*	
H22B_1	0.2642	0.3133	0.4905	0.107*	
H22C_1	0.1367	0.3201	0.5896	0.107*	
O1_2	0.32537 (14)	0.90895 (3)	0.94767 (12)	0.0394 (4)	
O2_2	0.36036 (14)	0.85187 (3)	0.92517 (12)	0.0393 (3)	
O3_2	0.60877 (18)	0.89453 (3)	0.72449 (15)	0.0632 (5)	
O4_2	0.20381 (16)	1.07102 (3)	0.95326 (13)	0.0498 (4)	
O5_2	0.45844 (18)	0.69115 (3)	0.84894 (15)	0.0636 (5)	
C1_2	0.4331 (2)	0.91701 (4)	0.86175 (17)	0.0349 (5)	
C2_2	0.5116 (2)	0.88934 (4)	0.80197 (19)	0.0400 (5)	
C3_2	0.4737 (2)	0.85542 (5)	0.83962 (18)	0.0360 (5)	
C4_2	0.3432 (2)	0.87840 (4)	1.01609 (18)	0.0355 (5)	
C5_2	0.2014 (2)	0.87194 (5)	1.0805 (2)	0.0542 (6)	
H5A_2	0.1239	0.8699	1.0126	0.081*	
H5B_2	0.1797	0.8903	1.1395	0.081*	
H5C_2	0.2083	0.8513	1.1312	0.081*	
C6_2	0.4701 (2)	0.88003 (5)	1.11275 (18)	0.0491 (6)	
H6A_2	0.5581	0.8854	1.0660	0.074*	
H6B_2	0.4824	0.8585	1.1566	0.074*	
H6C_2	0.4526	0.8971	1.1786	0.074*	
C7_2	0.4550 (2)	0.94842 (4)	0.82629 (17)	0.0369 (5)	
H7_2	0.5285	0.9512	0.7641	0.044*	
C8_2	0.3864 (2)	0.97912 (4)	0.86593 (18)	0.0340 (5)	
C9_2	0.2943 (2)	0.98221 (4)	0.97158 (18)	0.0375 (5)	
H9_2	0.2725	0.9631	1.0219	0.045*	
C10_2	0.2337 (2)	1.01245 (4)	1.00494 (19)	0.0389 (5)	
H10_2	0.1740	1.0140	1.0789	0.047*	
C11_2	0.2602 (2)	1.04031 (4)	0.93058 (19)	0.0369 (5)	
C12_2	0.3513 (2)	1.03770 (5)	0.82468 (19)	0.0436 (5)	
H12_2	0.3702	1.0567	0.7727	0.052*	
C13_2	0.4136 (2)	1.00793 (4)	0.79498 (18)	0.0394 (5)	
H13_2	0.4775	1.0069	0.7238	0.047*	
C14_2	0.1207 (3)	1.07459 (5)	1.0694 (2)	0.0588 (7)	
H14A_2	0.0875	1.0975	1.0766	0.088*	
H14B_2	0.1812	1.0689	1.1472	0.088*	
H14C_2	0.0367	1.0598	1.0636	0.088*	
C15_2	0.5357 (2)	0.82868 (4)	0.78873 (18)	0.0396 (5)	
H15_2	0.6068	0.8334	0.7261	0.047*	
C16_2	0.5129 (2)	0.79356 (5)	0.81271 (18)	0.0384 (5)	
C17_2	0.5856 (3)	0.77038 (5)	0.7371 (2)	0.0535 (6)	
H17_2	0.6500	0.7781	0.6729	0.064*	
C18_2	0.5669 (3)	0.73688 (5)	0.7525 (2)	0.0592 (7)	
H18_2	0.6186	0.7219	0.6994	0.071*	
C19_2	0.4736 (2)	0.72471 (5)	0.8443 (2)	0.0466 (6)	
C20_2	0.4040 (2)	0.74698 (5)	0.9235 (2)	0.0445 (5)	
H20_2	0.3425	0.7391	0.9895	0.053*	
C21_2	0.4227 (2)	0.78064 (5)	0.90765 (19)	0.0440 (5)	
H21_2	0.3728	0.7954	0.9628	0.053*	
C22_2	0.3599 (3)	0.67802 (5)	0.9400 (2)	0.0658 (7)	
H22A_2	0.3567	0.6539	0.9317	0.099*	
H22B_2	0.2631	0.6872	0.9213	0.099*	
H22C_2	0.3921	0.6840	1.0298	0.099*	

### Atomic displacement parameters (Å^2^)

**Table:**

	*U*^11^	*U*^22^	*U*^33^	*U*^12^	*U*^13^	*U*^23^
O1_1	0.0324 (9)	0.0299 (7)	0.0422 (8)	0.0016 (6)	−0.0044 (7)	−0.0061 (6)
O2_1	0.0329 (9)	0.0313 (7)	0.0472 (8)	−0.0001 (6)	−0.0053 (7)	0.0060 (6)
O3_1	0.0432 (11)	0.0466 (8)	0.0629 (10)	−0.0014 (7)	−0.0172 (9)	0.0026 (7)
O4_1	0.0548 (10)	0.0340 (8)	0.0533 (9)	0.0090 (7)	0.0024 (8)	−0.0062 (6)
O5_1	0.0754 (13)	0.0333 (8)	0.0974 (13)	−0.0039 (8)	−0.0303 (11)	0.0086 (8)
C1_1	0.0283 (13)	0.0357 (11)	0.0296 (10)	−0.0016 (9)	−0.0002 (10)	−0.0019 (8)
C2_1	0.0306 (13)	0.0377 (12)	0.0347 (11)	0.0003 (10)	−0.0048 (11)	0.0014 (9)
C3_1	0.0287 (13)	0.0342 (11)	0.0351 (11)	−0.0008 (9)	−0.0026 (10)	0.0012 (9)
C4_1	0.0337 (13)	0.0256 (10)	0.0356 (11)	0.0011 (9)	−0.0067 (10)	0.0012 (8)
C5_1	0.0423 (14)	0.0340 (11)	0.0620 (14)	−0.0005 (10)	−0.0151 (12)	−0.0016 (10)
C6_1	0.0534 (15)	0.0464 (12)	0.0360 (12)	−0.0008 (11)	0.0025 (11)	−0.0041 (10)
C7_1	0.0326 (12)	0.0361 (11)	0.0326 (11)	−0.0046 (9)	0.0014 (10)	−0.0018 (8)
C8_1	0.0308 (13)	0.0318 (10)	0.0314 (10)	−0.0028 (9)	0.0020 (10)	−0.0013 (8)
C9_1	0.0373 (14)	0.0328 (11)	0.0429 (12)	−0.0005 (9)	−0.0010 (11)	−0.0088 (9)
C10_1	0.0326 (13)	0.0396 (12)	0.0450 (12)	0.0035 (10)	0.0004 (11)	−0.0080 (9)
C11_1	0.0407 (14)	0.0302 (11)	0.0380 (12)	0.0018 (9)	0.0102 (11)	−0.0024 (9)
C12_1	0.0479 (15)	0.0341 (11)	0.0409 (12)	−0.0044 (10)	0.0002 (12)	−0.0068 (9)
C13_1	0.0394 (14)	0.0366 (11)	0.0359 (11)	−0.0034 (9)	−0.0011 (10)	−0.0022 (9)
C14_1	0.0503 (16)	0.0426 (12)	0.0661 (16)	0.0112 (11)	0.0045 (14)	0.0027 (11)
C15_1	0.0349 (13)	0.0379 (12)	0.0445 (12)	−0.0002 (10)	−0.0065 (10)	0.0058 (9)
C16_1	0.0376 (14)	0.0356 (11)	0.0438 (12)	0.0008 (10)	−0.0074 (11)	0.0065 (9)
C17_1	0.096 (2)	0.0414 (14)	0.102 (2)	−0.0100 (14)	−0.0660 (18)	0.0153 (13)
C18_1	0.105 (3)	0.0406 (15)	0.119 (2)	−0.0060 (15)	−0.074 (2)	0.0229 (14)
C19_1	0.0454 (15)	0.0329 (12)	0.0625 (15)	0.0001 (10)	−0.0108 (13)	0.0072 (10)
C20_1	0.0544 (16)	0.0376 (13)	0.0602 (15)	0.0036 (11)	−0.0202 (12)	0.0043 (11)
C21_1	0.0537 (16)	0.0347 (12)	0.0565 (14)	0.0059 (11)	−0.0147 (13)	0.0075 (10)
C22_1	0.069 (2)	0.0429 (14)	0.101 (2)	−0.0042 (13)	−0.0219 (17)	−0.0129 (13)
O1_2	0.0422 (9)	0.0335 (7)	0.0431 (8)	0.0003 (6)	0.0078 (7)	0.0037 (6)
O2_2	0.0422 (9)	0.0346 (7)	0.0414 (8)	−0.0036 (6)	0.0037 (7)	−0.0064 (6)
O3_2	0.0748 (12)	0.0501 (8)	0.0671 (10)	−0.0040 (8)	0.0366 (10)	−0.0040 (7)
O4_2	0.0613 (10)	0.0346 (8)	0.0542 (9)	0.0054 (7)	0.0112 (8)	0.0083 (6)
O5_2	0.0791 (12)	0.0335 (8)	0.0788 (11)	0.0083 (8)	0.0117 (10)	−0.0005 (7)
C1_2	0.0367 (13)	0.0365 (11)	0.0316 (11)	−0.0030 (9)	0.0023 (10)	0.0006 (9)
C2_2	0.0452 (14)	0.0377 (11)	0.0371 (12)	−0.0014 (10)	−0.0015 (11)	−0.0031 (9)
C3_2	0.0379 (13)	0.0392 (12)	0.0307 (11)	−0.0009 (10)	−0.0012 (10)	−0.0033 (9)
C4_2	0.0432 (14)	0.0260 (10)	0.0376 (11)	−0.0023 (9)	0.0047 (11)	−0.0020 (9)
C5_2	0.0527 (16)	0.0427 (12)	0.0687 (15)	−0.0015 (11)	0.0248 (13)	−0.0001 (10)
C6_2	0.0623 (17)	0.0491 (13)	0.0357 (12)	−0.0031 (11)	−0.0038 (12)	−0.0002 (10)
C7_2	0.0391 (13)	0.0397 (12)	0.0318 (11)	−0.0067 (9)	−0.0006 (10)	−0.0017 (9)
C8_2	0.0368 (13)	0.0314 (11)	0.0336 (11)	−0.0042 (9)	−0.0033 (10)	0.0020 (8)
C9_2	0.0423 (13)	0.0342 (11)	0.0360 (12)	−0.0042 (9)	−0.0004 (10)	0.0057 (9)
C10_2	0.0445 (14)	0.0340 (11)	0.0383 (12)	−0.0009 (10)	0.0030 (10)	0.0046 (9)
C11_2	0.0393 (13)	0.0310 (11)	0.0401 (12)	−0.0012 (9)	−0.0042 (11)	0.0015 (9)
C12_2	0.0505 (14)	0.0361 (12)	0.0443 (13)	−0.0066 (10)	0.0021 (11)	0.0093 (9)
C13_2	0.0436 (13)	0.0377 (11)	0.0369 (12)	−0.0065 (10)	0.0038 (11)	0.0024 (9)
C14_2	0.0650 (17)	0.0450 (13)	0.0681 (16)	0.0081 (12)	0.0236 (14)	0.0004 (11)
C15_2	0.0435 (14)	0.0407 (12)	0.0343 (11)	0.0030 (10)	−0.0011 (10)	−0.0037 (9)
C16_2	0.0396 (14)	0.0409 (12)	0.0340 (11)	0.0044 (10)	−0.0068 (10)	−0.0037 (9)
C17_2	0.0683 (17)	0.0409 (13)	0.0519 (14)	0.0055 (12)	0.0126 (13)	−0.0073 (10)
C18_2	0.0732 (19)	0.0417 (13)	0.0634 (16)	0.0142 (12)	0.0136 (14)	−0.0076 (11)
C19_2	0.0526 (15)	0.0330 (12)	0.0535 (14)	0.0092 (11)	−0.0086 (12)	−0.0029 (10)
C20_2	0.0501 (15)	0.0385 (12)	0.0447 (13)	0.0059 (10)	−0.0007 (11)	0.0007 (10)
C21_2	0.0489 (15)	0.0405 (12)	0.0426 (12)	0.0067 (10)	0.0000 (11)	−0.0074 (9)
C22_2	0.0771 (19)	0.0446 (13)	0.0756 (17)	0.0009 (13)	0.0013 (16)	0.0070 (12)

### Geometric parameters (Å, °)

**Table:**

O1_1—C1_1	1.377 (2)	O1_2—C1_2	1.386 (2)
O1_1—C4_1	1.427 (2)	O1_2—C4_2	1.423 (2)
O2_1—C3_1	1.377 (2)	O2_2—C3_2	1.392 (2)
O2_1—C4_1	1.429 (2)	O2_2—C4_2	1.427 (2)
O3_1—C2_1	1.237 (2)	O3_2—C2_2	1.233 (2)
O4_1—C11_1	1.376 (2)	O4_2—C11_2	1.368 (2)
O4_1—C14_1	1.421 (3)	O4_2—C14_2	1.438 (2)
O5_1—C19_1	1.375 (2)	O5_2—C19_2	1.363 (2)
O5_1—C22_1	1.412 (3)	O5_2—C22_2	1.423 (2)
C1_1—C7_1	1.343 (2)	C1_2—C7_2	1.336 (2)
C1_1—C2_1	1.476 (3)	C1_2—C2_2	1.474 (2)
C2_1—C3_1	1.474 (3)	C2_2—C3_2	1.468 (3)
C3_1—C15_1	1.336 (3)	C3_2—C15_2	1.335 (2)
C4_1—C5_1	1.493 (3)	C4_2—C6_2	1.505 (3)
C4_1—C6_1	1.505 (2)	C4_2—C5_2	1.508 (3)
C5_1—H5A_1	0.9800	C5_2—H5A_2	0.9800
C5_1—H5B_1	0.9800	C5_2—H5B_2	0.9800
C5_1—H5C_1	0.9800	C5_2—H5C_2	0.9800
C6_1—H6A_1	0.9800	C6_2—H6A_2	0.9800
C6_1—H6B_1	0.9800	C6_2—H6B_2	0.9800
C6_1—H6C_1	0.9800	C6_2—H6C_2	0.9800
C7_1—C8_1	1.461 (2)	C7_2—C8_2	1.456 (2)
C7_1—H7_1	0.9500	C7_2—H7_2	0.9500
C8_1—C9_1	1.391 (3)	C8_2—C13_2	1.396 (2)
C8_1—C13_1	1.397 (2)	C8_2—C9_2	1.399 (2)
C9_1—C10_1	1.392 (3)	C9_2—C10_2	1.390 (2)
C9_1—H9_1	0.9500	C9_2—H9_2	0.9500
C10_1—C11_1	1.376 (3)	C10_2—C11_2	1.382 (2)
C10_1—H10_1	0.9500	C10_2—H10_2	0.9500
C11_1—C12_1	1.387 (3)	C11_2—C12_2	1.393 (2)
C12_1—C13_1	1.380 (3)	C12_2—C13_2	1.371 (2)
C12_1—H12_1	0.9500	C12_2—H12_2	0.9500
C13_1—H13_1	0.9500	C13_2—H13_2	0.9500
C14_1—H14A_1	0.9800	C14_2—H14A_2	0.9800
C14_1—H14B_1	0.9800	C14_2—H14B_2	0.9800
C14_1—H14C_1	0.9800	C14_2—H14C_2	0.9800
C15_1—C16_1	1.459 (3)	C15_2—C16_2	1.455 (3)
C15_1—H15_1	0.9500	C15_2—H15_2	0.9500
C16_1—C17_1	1.376 (3)	C16_2—C21_2	1.398 (2)
C16_1—C21_1	1.379 (3)	C16_2—C17_2	1.398 (2)
C17_1—C18_1	1.376 (3)	C17_2—C18_2	1.373 (3)
C17_1—H17_1	0.9500	C17_2—H17_2	0.9500
C18_1—C19_1	1.363 (3)	C18_2—C19_2	1.383 (3)
C18_1—H18_1	0.9500	C18_2—H18_2	0.9500
C19_1—C20_1	1.361 (3)	C19_2—C20_2	1.380 (2)
C20_1—C21_1	1.390 (3)	C20_2—C21_2	1.380 (3)
C20_1—H20_1	0.9500	C20_2—H20_2	0.9500
C21_1—H21_1	0.9500	C21_2—H21_2	0.9500
C22_1—H22A_1	0.9800	C22_2—H22A_2	0.9800
C22_1—H22B_1	0.9800	C22_2—H22B_2	0.9800
C22_1—H22C_1	0.9800	C22_2—H22C_2	0.9800
			
C1_1—O1_1—C4_1	116.33 (14)	C1_2—O1_2—C4_2	115.98 (14)
C3_1—O2_1—C4_1	115.59 (14)	C3_2—O2_2—C4_2	115.48 (13)
C11_1—O4_1—C14_1	116.85 (16)	C11_2—O4_2—C14_2	116.54 (14)
C19_1—O5_1—C22_1	117.70 (18)	C19_2—O5_2—C22_2	117.48 (16)
C7_1—C1_1—O1_1	121.09 (17)	C7_2—C1_2—O1_2	120.79 (16)
C7_1—C1_1—C2_1	121.51 (19)	C7_2—C1_2—C2_2	121.82 (16)
O1_1—C1_1—C2_1	117.13 (16)	O1_2—C1_2—C2_2	117.12 (15)
O3_1—C2_1—C3_1	121.07 (18)	O3_2—C2_2—C3_2	120.72 (17)
O3_1—C2_1—C1_1	121.52 (18)	O3_2—C2_2—C1_2	120.91 (16)
C3_1—C2_1—C1_1	117.40 (19)	C3_2—C2_2—C1_2	118.35 (16)
C15_1—C3_1—O2_1	120.54 (18)	C15_2—C3_2—O2_2	120.08 (17)
C15_1—C3_1—C2_1	122.7 (2)	C15_2—C3_2—C2_2	122.99 (17)
O2_1—C3_1—C2_1	116.61 (17)	O2_2—C3_2—C2_2	116.76 (16)
O1_1—C4_1—O2_1	109.36 (14)	O1_2—C4_2—O2_2	110.35 (14)
O1_1—C4_1—C5_1	106.03 (14)	O1_2—C4_2—C6_2	110.84 (16)
O2_1—C4_1—C5_1	105.43 (15)	O2_2—C4_2—C6_2	110.73 (16)
O1_1—C4_1—C6_1	110.76 (14)	O1_2—C4_2—C5_2	105.92 (16)
O2_1—C4_1—C6_1	110.87 (15)	O2_2—C4_2—C5_2	105.59 (15)
C5_1—C4_1—C6_1	114.12 (16)	C6_2—C4_2—C5_2	113.18 (17)
C4_1—C5_1—H5A_1	109.5	C4_2—C5_2—H5A_2	109.5
C4_1—C5_1—H5B_1	109.5	C4_2—C5_2—H5B_2	109.5
H5A_1—C5_1—H5B_1	109.5	H5A_2—C5_2—H5B_2	109.5
C4_1—C5_1—H5C_1	109.5	C4_2—C5_2—H5C_2	109.5
H5A_1—C5_1—H5C_1	109.5	H5A_2—C5_2—H5C_2	109.5
H5B_1—C5_1—H5C_1	109.5	H5B_2—C5_2—H5C_2	109.5
C4_1—C6_1—H6A_1	109.5	C4_2—C6_2—H6A_2	109.5
C4_1—C6_1—H6B_1	109.5	C4_2—C6_2—H6B_2	109.5
H6A_1—C6_1—H6B_1	109.5	H6A_2—C6_2—H6B_2	109.5
C4_1—C6_1—H6C_1	109.5	C4_2—C6_2—H6C_2	109.5
H6A_1—C6_1—H6C_1	109.5	H6A_2—C6_2—H6C_2	109.5
H6B_1—C6_1—H6C_1	109.5	H6B_2—C6_2—H6C_2	109.5
C1_1—C7_1—C8_1	130.2 (2)	C1_2—C7_2—C8_2	131.48 (17)
C1_1—C7_1—H7_1	114.9	C1_2—C7_2—H7_2	114.3
C8_1—C7_1—H7_1	114.9	C8_2—C7_2—H7_2	114.3
C9_1—C8_1—C13_1	116.77 (18)	C13_2—C8_2—C9_2	116.59 (17)
C9_1—C8_1—C7_1	124.86 (17)	C13_2—C8_2—C7_2	118.59 (16)
C13_1—C8_1—C7_1	118.38 (19)	C9_2—C8_2—C7_2	124.82 (16)
C8_1—C9_1—C10_1	121.72 (18)	C10_2—C9_2—C8_2	121.77 (17)
C8_1—C9_1—H9_1	119.1	C10_2—C9_2—H9_2	119.1
C10_1—C9_1—H9_1	119.1	C8_2—C9_2—H9_2	119.1
C11_1—C10_1—C9_1	120.0 (2)	C11_2—C10_2—C9_2	120.06 (17)
C11_1—C10_1—H10_1	120.0	C11_2—C10_2—H10_2	120.0
C9_1—C10_1—H10_1	120.0	C9_2—C10_2—H10_2	120.0
O4_1—C11_1—C10_1	124.5 (2)	O4_2—C11_2—C10_2	124.74 (16)
O4_1—C11_1—C12_1	115.93 (18)	O4_2—C11_2—C12_2	116.28 (16)
C10_1—C11_1—C12_1	119.56 (18)	C10_2—C11_2—C12_2	118.98 (17)
C13_1—C12_1—C11_1	119.87 (19)	C13_2—C12_2—C11_2	120.41 (17)
C13_1—C12_1—H12_1	120.1	C13_2—C12_2—H12_2	119.8
C11_1—C12_1—H12_1	120.1	C11_2—C12_2—H12_2	119.8
C12_1—C13_1—C8_1	122.1 (2)	C12_2—C13_2—C8_2	122.14 (17)
C12_1—C13_1—H13_1	119.0	C12_2—C13_2—H13_2	118.9
C8_1—C13_1—H13_1	119.0	C8_2—C13_2—H13_2	118.9
O4_1—C14_1—H14A_1	109.5	O4_2—C14_2—H14A_2	109.5
O4_1—C14_1—H14B_1	109.5	O4_2—C14_2—H14B_2	109.5
H14A_1—C14_1—H14B_1	109.5	H14A_2—C14_2—H14B_2	109.5
O4_1—C14_1—H14C_1	109.5	O4_2—C14_2—H14C_2	109.5
H14A_1—C14_1—H14C_1	109.5	H14A_2—C14_2—H14C_2	109.5
H14B_1—C14_1—H14C_1	109.5	H14B_2—C14_2—H14C_2	109.5
C3_1—C15_1—C16_1	129.6 (2)	C3_2—C15_2—C16_2	131.06 (18)
C3_1—C15_1—H15_1	115.2	C3_2—C15_2—H15_2	114.5
C16_1—C15_1—H15_1	115.2	C16_2—C15_2—H15_2	114.5
C17_1—C16_1—C21_1	115.4 (2)	C21_2—C16_2—C17_2	116.04 (17)
C17_1—C16_1—C15_1	119.5 (2)	C21_2—C16_2—C15_2	124.91 (16)
C21_1—C16_1—C15_1	125.10 (19)	C17_2—C16_2—C15_2	119.04 (17)
C18_1—C17_1—C16_1	122.9 (2)	C18_2—C17_2—C16_2	122.16 (19)
C18_1—C17_1—H17_1	118.6	C18_2—C17_2—H17_2	118.9
C16_1—C17_1—H17_1	118.6	C16_2—C17_2—H17_2	118.9
C19_1—C18_1—C17_1	120.3 (2)	C17_2—C18_2—C19_2	120.72 (19)
C19_1—C18_1—H18_1	119.8	C17_2—C18_2—H18_2	119.6
C17_1—C18_1—H18_1	119.8	C19_2—C18_2—H18_2	119.6
C20_1—C19_1—C18_1	118.8 (2)	O5_2—C19_2—C20_2	125.26 (19)
C20_1—C19_1—O5_1	125.3 (2)	O5_2—C19_2—C18_2	116.33 (17)
C18_1—C19_1—O5_1	115.9 (2)	C20_2—C19_2—C18_2	118.41 (18)
C19_1—C20_1—C21_1	120.1 (2)	C19_2—C20_2—C21_2	120.74 (19)
C19_1—C20_1—H20_1	119.9	C19_2—C20_2—H20_2	119.6
C21_1—C20_1—H20_1	119.9	C21_2—C20_2—H20_2	119.6
C16_1—C21_1—C20_1	122.4 (2)	C20_2—C21_2—C16_2	121.86 (17)
C16_1—C21_1—H21_1	118.8	C20_2—C21_2—H21_2	119.1
C20_1—C21_1—H21_1	118.8	C16_2—C21_2—H21_2	119.1
O5_1—C22_1—H22A_1	109.5	O5_2—C22_2—H22A_2	109.5
O5_1—C22_1—H22B_1	109.5	O5_2—C22_2—H22B_2	109.5
H22A_1—C22_1—H22B_1	109.5	H22A_2—C22_2—H22B_2	109.5
O5_1—C22_1—H22C_1	109.5	O5_2—C22_2—H22C_2	109.5
H22A_1—C22_1—H22C_1	109.5	H22A_2—C22_2—H22C_2	109.5
H22B_1—C22_1—H22C_1	109.5	H22B_2—C22_2—H22C_2	109.5
			
C4_1—O1_1—C1_1—C7_1	−165.65 (15)	C4_2—O1_2—C1_2—C7_2	−157.74 (19)
C4_1—O1_1—C1_1—C2_1	20.2 (2)	C4_2—O1_2—C1_2—C2_2	28.1 (2)
C7_1—C1_1—C2_1—O3_1	16.9 (3)	C7_2—C1_2—C2_2—O3_2	5.9 (3)
O1_1—C1_1—C2_1—O3_1	−168.91 (16)	O1_2—C1_2—C2_2—O3_2	179.93 (19)
C7_1—C1_1—C2_1—C3_1	−162.07 (16)	C7_2—C1_2—C2_2—C3_2	−175.7 (2)
O1_1—C1_1—C2_1—C3_1	12.1 (2)	O1_2—C1_2—C2_2—C3_2	−1.6 (3)
C4_1—O2_1—C3_1—C15_1	157.51 (16)	C4_2—O2_2—C3_2—C15_2	153.85 (19)
C4_1—O2_1—C3_1—C2_1	−26.6 (2)	C4_2—O2_2—C3_2—C2_2	−30.8 (2)
O3_1—C2_1—C3_1—C15_1	−12.0 (3)	O3_2—C2_2—C3_2—C15_2	−3.3 (3)
C1_1—C2_1—C3_1—C15_1	167.01 (17)	C1_2—C2_2—C3_2—C15_2	178.3 (2)
O3_1—C2_1—C3_1—O2_1	172.26 (16)	O3_2—C2_2—C3_2—O2_2	−178.54 (19)
C1_1—C2_1—C3_1—O2_1	−8.7 (2)	C1_2—C2_2—C3_2—O2_2	3.0 (3)
C1_1—O1_1—C4_1—O2_1	−54.47 (19)	C1_2—O1_2—C4_2—O2_2	−55.1 (2)
C1_1—O1_1—C4_1—C5_1	−167.69 (14)	C1_2—O1_2—C4_2—C6_2	67.95 (19)
C1_1—O1_1—C4_1—C6_1	68.01 (19)	C1_2—O1_2—C4_2—C5_2	−168.92 (16)
C3_1—O2_1—C4_1—O1_1	58.03 (18)	C3_2—O2_2—C4_2—O1_2	56.5 (2)
C3_1—O2_1—C4_1—C5_1	171.65 (14)	C3_2—O2_2—C4_2—C6_2	−66.6 (2)
C3_1—O2_1—C4_1—C6_1	−64.38 (19)	C3_2—O2_2—C4_2—C5_2	170.52 (16)
O1_1—C1_1—C7_1—C8_1	−0.5 (3)	O1_2—C1_2—C7_2—C8_2	2.7 (3)
C2_1—C1_1—C7_1—C8_1	173.45 (16)	C2_2—C1_2—C7_2—C8_2	176.5 (2)
C1_1—C7_1—C8_1—C9_1	5.3 (3)	C1_2—C7_2—C8_2—C13_2	−168.8 (2)
C1_1—C7_1—C8_1—C13_1	−174.35 (17)	C1_2—C7_2—C8_2—C9_2	11.6 (4)
C13_1—C8_1—C9_1—C10_1	0.0 (3)	C13_2—C8_2—C9_2—C10_2	−0.4 (3)
C7_1—C8_1—C9_1—C10_1	−179.61 (15)	C7_2—C8_2—C9_2—C10_2	179.2 (2)
C8_1—C9_1—C10_1—C11_1	0.3 (3)	C8_2—C9_2—C10_2—C11_2	2.0 (3)
C14_1—O4_1—C11_1—C10_1	10.7 (2)	C14_2—O4_2—C11_2—C10_2	5.2 (3)
C14_1—O4_1—C11_1—C12_1	−169.49 (16)	C14_2—O4_2—C11_2—C12_2	−174.45 (19)
C9_1—C10_1—C11_1—O4_1	179.90 (15)	C9_2—C10_2—C11_2—O4_2	178.6 (2)
C9_1—C10_1—C11_1—C12_1	0.1 (3)	C9_2—C10_2—C11_2—C12_2	−1.7 (3)
O4_1—C11_1—C12_1—C13_1	179.41 (15)	O4_2—C11_2—C12_2—C13_2	179.6 (2)
C10_1—C11_1—C12_1—C13_1	−0.8 (3)	C10_2—C11_2—C12_2—C13_2	−0.1 (3)
C11_1—C12_1—C13_1—C8_1	1.1 (3)	C11_2—C12_2—C13_2—C8_2	1.7 (3)
C9_1—C8_1—C13_1—C12_1	−0.7 (3)	C9_2—C8_2—C13_2—C12_2	−1.4 (3)
C7_1—C8_1—C13_1—C12_1	178.93 (15)	C7_2—C8_2—C13_2—C12_2	178.9 (2)
O2_1—C3_1—C15_1—C16_1	−6.8 (3)	O2_2—C3_2—C15_2—C16_2	−4.7 (3)
C2_1—C3_1—C15_1—C16_1	177.61 (16)	C2_2—C3_2—C15_2—C16_2	−179.8 (2)
C3_1—C15_1—C16_1—C17_1	171.0 (2)	C3_2—C15_2—C16_2—C21_2	−5.3 (4)
C3_1—C15_1—C16_1—C21_1	−11.7 (3)	C3_2—C15_2—C16_2—C17_2	174.9 (2)
C21_1—C16_1—C17_1—C18_1	1.1 (4)	C21_2—C16_2—C17_2—C18_2	1.6 (3)
C15_1—C16_1—C17_1—C18_1	178.6 (2)	C15_2—C16_2—C17_2—C18_2	−178.6 (2)
C16_1—C17_1—C18_1—C19_1	−0.5 (5)	C16_2—C17_2—C18_2—C19_2	0.3 (4)
C17_1—C18_1—C19_1—C20_1	−0.8 (4)	C22_2—O5_2—C19_2—C20_2	1.9 (3)
C17_1—C18_1—C19_1—O5_1	178.9 (2)	C22_2—O5_2—C19_2—C18_2	−178.2 (2)
C22_1—O5_1—C19_1—C20_1	−3.6 (3)	C17_2—C18_2—C19_2—O5_2	177.7 (2)
C22_1—O5_1—C19_1—C18_1	176.8 (2)	C17_2—C18_2—C19_2—C20_2	−2.4 (4)
C18_1—C19_1—C20_1—C21_1	1.4 (3)	O5_2—C19_2—C20_2—C21_2	−177.6 (2)
O5_1—C19_1—C20_1—C21_1	−178.2 (2)	C18_2—C19_2—C20_2—C21_2	2.5 (3)
C17_1—C16_1—C21_1—C20_1	−0.5 (3)	C19_2—C20_2—C21_2—C16_2	−0.6 (3)
C15_1—C16_1—C21_1—C20_1	−177.87 (18)	C17_2—C16_2—C21_2—C20_2	−1.5 (3)
C19_1—C20_1—C21_1—C16_1	−0.7 (3)	C15_2—C16_2—C21_2—C20_2	178.8 (2)

### Hydrogen-bond geometry (Å, °)

**Table:**

Cg(II_2) is the centroid of ring II (C8–C13) of molecule 2.

**Table:**

*D*—H···*A*	*D*—H	H···*A*	*D*···*A*	*D*—H···*A*
C13—H13···Cg(II_2)^i^	0.95	2.68	3.604 (2)	164

Symmetry codes: (i) −*x*, −*y*+1, −*z*+1.

## References

[bb1] Abaee, M. S., Massa, W., Mojtahedi, M. M. & Mesbah, A. W. (2012). *Acta Cryst.* E**68**, o355. 10.1107/S1600536811055632 PMC3275037 22346982

[bb2] Abaee, M. S., Mojtahedi, M. M., Hamidi, V., Mesbah, A. W. & Massa, W. (2008*a*). *Synthesis*, pp. 2122–2126.

[bb3] Abaee, M. S., Mojtahedi, M. M., Sharifi, R., Zahedi, M. M., Mesbah, A. W. & Massa, W. (2008*b*). *J. Chem. Res.* pp. 388–389.

[bb4] Blessing, R. H. (1995). *Acta Cryst.* A**51**, 33–38. 10.1107/s0108767394005726 7702794

[bb5] Brandenburg, K. (2011). *DIAMOND* Crystal Impact GbR, Bonn, Germany.

[bb6] Hunter, C. A. & Sanders, J. K. M. (1990). *J. Am. Chem. Soc.* **112**, 5525–5534.

[bb7] Nesterov, V. V., Sarkisov, S. S., Shulaev, V. & Nesterov, V. N. (2011). *Acta Cryst.* E**67**, o760–o761. 10.1107/S1600536811006994 PMC3099892 21754057

[bb8] Shahani, T., Fun, H.-K., Balaji, G. L., Vijayakumar, V. & Sarveswari, S. (2010). *Acta Cryst.* E**66**, o630–o631. 10.1107/S1600536810005192 PMC2983730 21580387

[bb9] Sheldrick, G. M. (2008). *Acta Cryst.* A**64**, 112–122. 10.1107/S0108767307043930 18156677

[bb10] Stoe & Cie (1999). *EXPOSE*, *CELL* and *INTEGRATE* in *IPDSI Software* Stoe & Cie, Darmstadt, Germany.

[bb11] Westrip, S. P. (2010). *J. Appl. Cryst.* **43**, 920–925.

